# The lncRNA ZFAS1 regulates lipogenesis in colorectal cancer by binding polyadenylate-binding protein 2 to stabilize SREBP1 mRNA

**DOI:** 10.1016/j.omtn.2021.12.010

**Published:** 2021-12-11

**Authors:** Huishan Wang, Yuli Chen, Yanwen Liu, Qiuhui Li, Jing Luo, Li Wang, Yuanyuan Chen, Chen Sang, Wen Zhang, Xianxiu Ge, Zhifeng Yao, Lin Miao, Xianghua Liu

**Affiliations:** 1 Department of Gastroenterology, Shanghai Songjiang District Central Hospital, Shanghai 210000, China; 2 Department of Biochemistry and Molecular Biology, Nanjing Medical University, Nanjing, China; 3 Department of Oncology, Zhongda Hospital, Medical School of Southeast University, Nanjing 210000, China; 4 Department of Cardiothoracic Surgery, Jinling Hospital, Medical School of Nanjing University, Nanjing 210011, China; 5 Department of Oncology, The Third Medical School of Nanjing Medical University, Nanjing 210011, China; 6 Department of Cardiothoracic Surgery, The Third Affiliated Hospital of Soochow of University, Changzhou G 213003, China; 7 Medical Center for Digestive Diseases, The Second Affiliated Hospital of Nanjing Medical University, Nanjing 210011, China

**Keywords:** lncRNA, CRC, ZFAS1, SREBP1, lipogenesis

## Abstract

Colorectal cancer (CRC) is the fourth leading cause of cancer-related mortality globally. Therefore, a better understanding of the early molecular events of this disease is needed. Long noncoding RNAs (lncRNAs) play a critical role in the regulation of tumorigenesis and cancer progression. In this study, we investigated the characteristics of ZFAS1 in CRC. We analyzed three independent microarray datasets of CRC tissues from GEO and found that ZFAS1 expression was remarkably upregulated in all three datasets. Moreover, we validated the overexpression of ZFAS1 in CRC tissues compared with normal tissues and found that ZFAS1 was positively correlated with tumor size and metastasis in CRC. Knockdown of ZFAS1 significantly suppressed the malignant phenotype and lipogenesis of CRC cells. Mechanistically, ZFAS1 binds polyadenylate-binding protein 2 (PABP2) to stabilize SREBP1 mRNA, thereby increasing the expression of SREBP1 and its target genes stearoyl-CoA desaturase (SCD1) and fatty acid synthase (FASN), thus promoting CRC lipid accumulation. These data demonstrated that ZFAS1 could act as an oncogene for CRC and that ZFAS1 reprograms lipid metabolism by binding with PABP2 to stabilize SREBP1 mRNA accumulation, implicating it as a novel and potent target for the treatment of CRC.

## Graphical abstract

**Figure:**
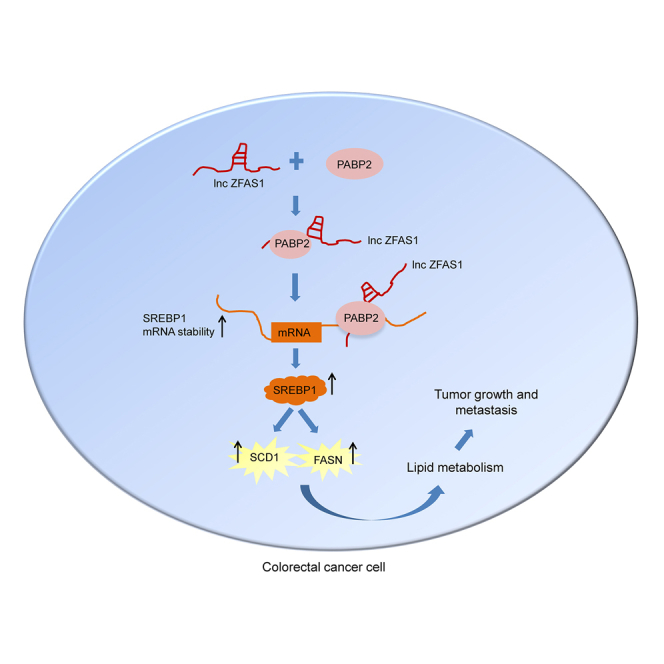

## Teaser

Colorectal cancer (CRC) is a leading cause of cancer-related death worldwide. Liu et al. report that long noncoding RNA ZFAS1 regulates fatty acid synthesis, thereby providing survival advantages for malignant phenotype transformation of CRC, and may be a potential therapeutic target for CRC.

## Introduction

Colorectal cancer (CRC) is the fourth most common malignant tumor in the world, with almost 900,000 deaths annually, and is mainly caused by distant metastasis.^1^ In addition, a high-fat diet increases the incidence of colorectal cancer. The hallmarks of cancer include proliferation, apoptosis, metastasis, abnormal metabolism, and angiogenesis.^2^ Recently, mounting evidence has shown that the metabolic reprogramming of tumor cells plays a vital role in tumorigenesis and progression.^3^ For example, aerobic glycolysis and increased glutamine metabolism are crucial for cancer cells to be shed from a primary tumor, to overcome nutrient and energy deficits, and eventually survive and form metastases.^4^^,^^5^ Therefore, a better understanding of the early metabolic events associated with the CRC malignant phenotype is warranted to decrease mortality and prolong patient survival.

A growing number of recent studies have shown that lipid metabolism, which confers the aggressive properties of malignant cancers, in particular the uncontrolled *de novo* synthesis of fatty acids (FAs), continues to provide the necessary building block molecules and participate in cell signal transduction as a second messenger, giving tumor cells growth and survival advantages.^6^, ^7^, ^8^, ^9^ The induction of lipogenesis is mainly controlled by sterol regulatory element-binding protein 1 (SREBP1), which is an ER membrane-bound protein that functions as a transcription factor for many key enzymes, such as stearoyl-CoA desaturase (SCD1) and fatty acid synthase (FASN).^10^ Moreover, overexpression of SREBP1 predicts poor prognosis and aggressive tumor biology.^11^^,^^12^ In addition, dysregulation of lipid metabolism has been found to contribute to the development of CRC and can be used to evaluate prognosis.^13^^,^^14^ Consequently, identifying a reliable molecule participating in the regulation of lipid metabolism and progression of CRC is urgently needed.

Long noncoding RNAs (lncRNAs) are a class of RNA molecules >200 nt in length with limited or no protein-coding capacity. Researchers have demonstrated that aberrant lncRNA expression is involved in diverse human diseases, particularly cancers.^15^^,^^16^ Increasing studies show that lncRNA expression is frequently dysregulated in various types of cancers, and some lncRNAs are correlated with cancer recurrence and poor prognosis.^17^ Mounting evidence has shown that lncRNAs are involved in metastasis, proliferation, cell differentiation, immune response, tumor metabolism, and other processes in tumors.^18^, ^19^, ^20^ These lncRNAs repress tumor suppressors or active oncogenes via different mechanisms, including epigenetic modification, alternative splicing, RNA decay, and posttranslational modification regulation.^21^^,^^22^ For instance, SNHG7 acts as a sponge for miR-216b to promote proliferation and liver metastasis of colorectal cancer by upregulating GALNT1^23^; HULC modulates abnormal lipid metabolism in hepatoma cells through the miR-9-mediated RXRA signaling pathway.^24^

ZNFX1 antisense RNA1 (ZFAS1) is overexpressed in a variety of cancers, including CRC.^25^ Several studies have shown that ZFAS1 functions as an oncogene by regulating miRNA and plays a vital role in the proliferation and metastasis of CRC.^26^ However, the molecular mechanisms of ZFAS1 involved in metabolic reprogramming remain largely unknown. In this study, we found that ZFAS1 expression was remarkably upregulated in CRC tissues and was positively correlated with advanced pathological stages and larger tumor sizes. In addition, knockdown of ZFAS1 significantly suppressed the malignant phenotype and lipogenesis in CRC cells. Mechanistically, an RNA pulldown assay demonstrated that ZFAS1 could bind to polyadenylate-binding protein 2 (PABP2), which is required for progressive and efficient polymerization of poly(A) tails at the 3′ ends of eukaryotic transcripts and control of the stability of bulk mRNA.^27^^,^^28^ RNA immunoprecipitation (RIP) assays indicated that ZFAS1 enhanced PABP2 binding with SREBP1 mRNA, which stabilizes SREBP1 mRNA and augments transcription of SCD1 and FASN, finally leading to accelerated lipid accumulation and promotion of the malignant phenotype of colorectal cancer cells. In conclusion, our data demonstrate that ZFAS1 regulates fatty acid synthesis, thereby providing survival advantages for malignant phenotype transformation of CRC and may be a potential therapeutic target for CRC.

## Results

### ZFAS1 is overexpressed in CRC and associated with poor prognosis

**Figure 1:**
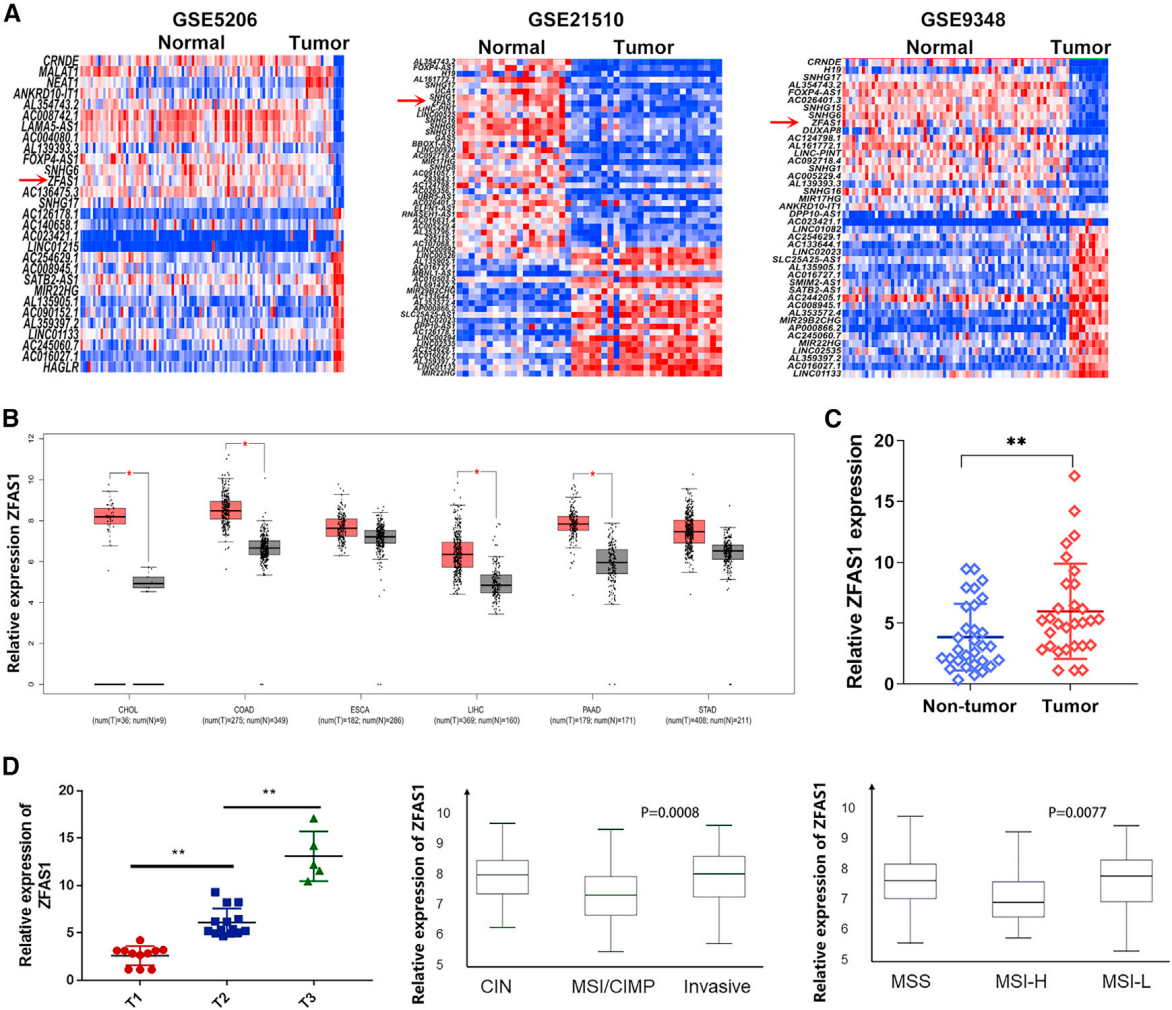
ZFAS1 is overexpressed in CRC tissues and correlates with pathological parameters (A) The differentially expressed lncRNAs in colorectal cancer tissues compared with normal lung tissues in the GSE5206, GSE21510, and GSE9348 datasets. (B) The differentially expressed lncRNAs in cholangiocarcinoma, colon adenocarcinoma, esophageal carcinoma, liver hepatocellular carcinoma, pancreatic adenocarcinoma, and stomach adenocarcinoma tissues compared with normal tissues in the TCGA dataset. (C) Relative expression of ZFAS1 in tumor tissue compared with adjacent normal tissues (n = 30). ZFAS1 expression was evaluated by qRT-PCR and normalized to GAPDH expression. (D) High expression of ZFAS1 was correlated with more advanced tumor size, invasive status, and microsatellite stability. ∗p < 0.05, ∗∗p < 0.01.

To screen for aberrantly expressed lncRNAs in CRC, we downloaded three CRC tissue microarray datasets (GSE5206, GSE21510, and GSE9348) from the Gene Expression Omnibus (GEO) and analyzed the differentially expressed lncRNAs. Among these dysregulated lncRNAs, ZFAS1 was the only lncRNA that was overexpressed in CRC tissues compared with noncancerous tissues in all three datasets (Figure 1A). ZFAS1 was therefore chosen as a candidate in this study.

Further analysis of The Cancer Genome Atlas (TCGA) data revealed that ZFAS1 was upregulated in CRC among the six digestive system tumors esophageal carcinoma (ESCA), esophageal carcinoma (STAD), colon adenocarcinoma (COAD), cholangio carcinoma (CHOL), pancreatic adenocarcinoma (PAAD), and liver hepatocellular carcinoma (LIHC) (Figure 1B). Next, we detected ZFAS1 expression in 30 paired CRC samples and adjacent tissues, and the qRT-PCR results showed that ZFAS1 expression was significantly overexpressed in CRC (p = 0.0001) compared with adjacent normal tissues (Figure 1C). Based on the relative ZFAS1 expression in tumor tissues, the 30 CRC patients were classified into two groups: the high-ZFAS1 group (n = 20, fold change ≥2) and the low-ZFAS1 group (n = 10, fold change < 2). Notably, high ZFAS1 expression in CRC was significantly correlated with tumor size, invasive status (p = 0.0008), and microsatellite stability (p = 0.0077) (Figure 1D). These results implied that ZFAS1 might act as an oncogene to promote the progression of CRC and might provide imperative clinical significance in CRC.

### Downregulation of ZFAS1 inhibits cell proliferation and invasion in CRC cells

**Figure 2:**
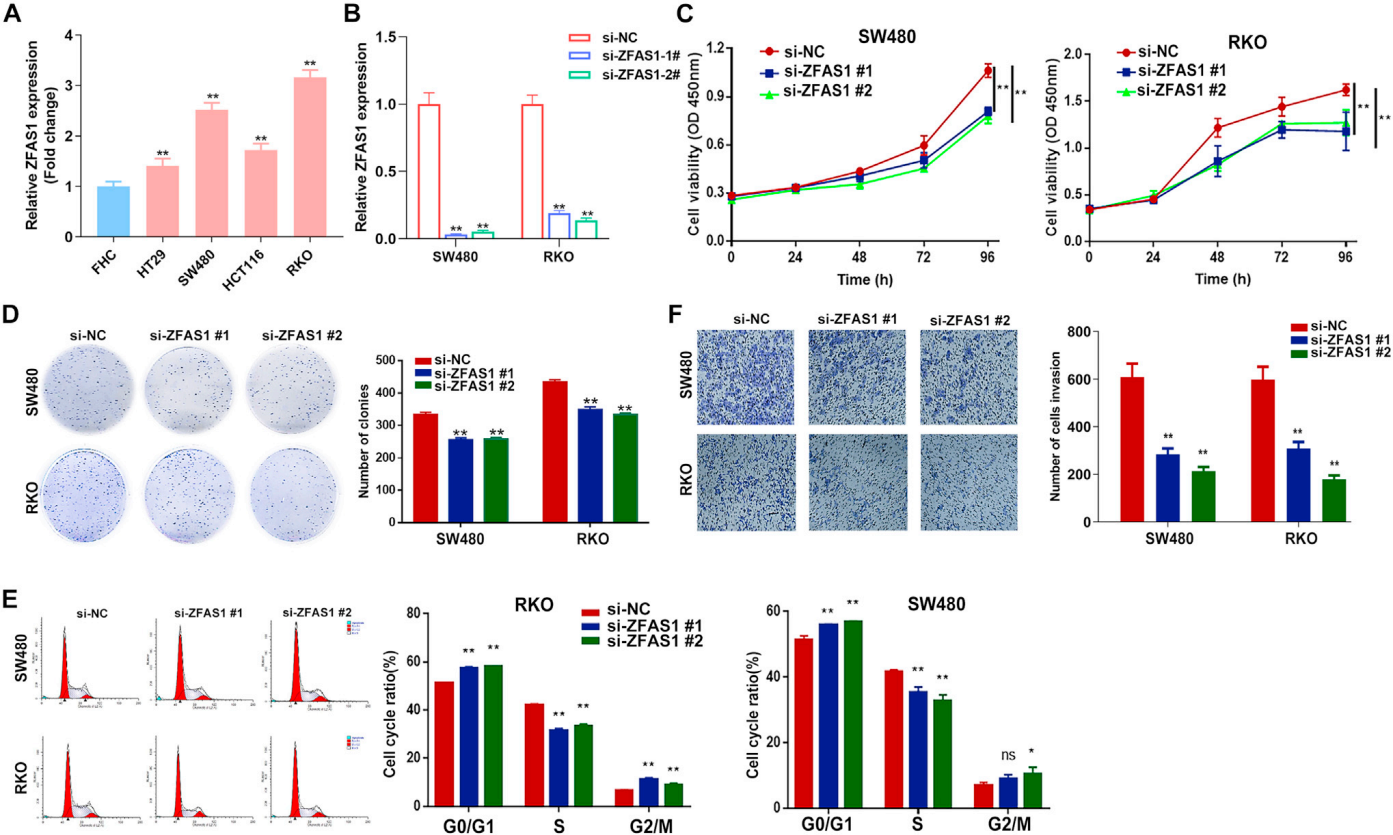
ZFAS1 promoted the CRC cell malignant phenotype *in vitro* (A) The expression of ZFAS1 in colorectal cancer cell lines (SW480, RKO, HT-29, HCT116) and normal colonic epithelial cells (FHC) was detected by qRT-PCR. (B) Two specific siRNAs were transfected into SW480 and RKO cell lines and effectively downregulated the expression of ZFAS1 compared with the negative control (Si-NC). (C) The CCK-8 assay showed that knockdown of ZFAS1 inhibited the growth of SW480 and RKO cell lines. (D) The colony formation assay showed that the colony numbers of the si-ZFAS1 groups were less than those of the control group. (E) Knockdown of ZFAS1 induced G1/S cell-cycle arrest. (F) The Transwell assay showed that the invasive numbers of the si-ZFAS1 groups were less than those of the control group. Three independent experiments were performed. Data are shown as the mean ± SD. ∗p < 0.05; ∗∗p < 0.01; ns, not significant.

To investigate the effect of ZFAS1 on CRC cells, we first examined the endogenous expression levels of ZFAS1 in various cancer cell lines by qRT-PCR. As shown in Figure 2A, of the five CRC cell lines (FHC, HT-29, SW480, RKO, and HCT116), SW480 and RKO cells expressed higher levels of ZFAS1 than human normal colonic epithelial cells (FHCs). Then, we performed loss-of-function experiments in SW480 and RKO cells. The results showed that ZFAS1 expression was effectively knocked down in RKO and SW480 cells by two different small interfering RNAs (siRNAs) (Figure 2B), which were subsequently used in further experiments. Furthermore, the results of the Cell Counting Kit-8 (CCK-8) assay and colony formation assay showed that knockdown of ZFAS1 inhibited CRC cell proliferation and colony formation capacity (Figures 2C and 2D). In addition, downregulation of ZFAS1 could induce G1/S cell-cycle arrest in SW480 and RKO cells (Figure 2E). To investigate the effect of ZFAS1 on CRC cell invasion, Transwell assays were performed, and the results revealed that inhibition of ZFAS1 decreased RKO and SW480 cell invasive ability (Figure 2F). These results indicate that ZFAS1 plays important roles in the malignant phenotype of CRC cells.

### ZFAS1 modulates lipid metabolism in CRC cells by regulating SREBP1

**Figure 3:**
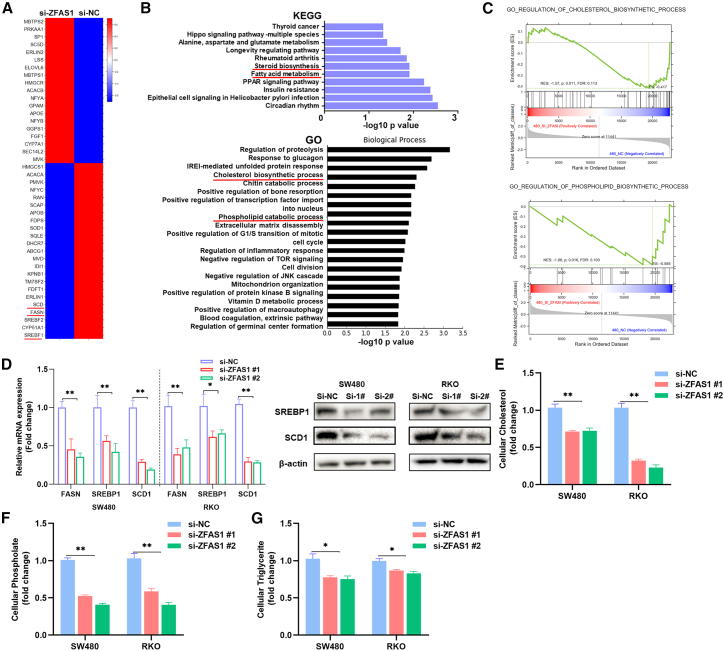
ZFAS1 is involved in lipid metabolism in CRC cells (A) Heatmap of the differentially expressed genes that were analyzed by next-generation sequencing in ZFAS1-downregulated SW480 cells and control cells. (B) KEGG pathway and Gene Ontology analysis of the differentially expressed genes presented by −Log 10(p value). Red lines underline cholesterol biosynthetic processes and phospholipid catabolic processes. (C) GSEA was performed to analyze the enrichment of differentially expressed genes. (D) qRT-PCR and western blot analysis of SREBP1, SCD1, and FASN expression levels in ZFAS1 siRNAs and negative control siRNA-transfected SW480 and RKO cells. (E–G) The cellular levels of cholesterol, phospholipids and triglycerides were examined with corresponding kits in SW480 and RKO cell lines after transfection with si-ZFAS1 or si-NC. ∗p < 0.05; ∗∗p < 0.01.

Next, we explored how ZFAS1 was involved in the progression of CRC. First, we conducted next-generation sequencing in SW480 cells after knockdown of ZFAS1 and found that the expression of 2,165 genes (p < 0.05, fold change >2) was upregulated or downregulated in ZFAS1 knockdown cells compared with control cells (Table S2; Figure 3A). Then, we performed pathway enrichment analysis of these differentially expressed genes and found that these genes are involved in steroid biosynthesis and fatty acid metabolism in CRC (Figure 3B). Gene set enrichment analysis (GSEA) also showed that ZFAS1-regulated genes were enriched in cholesterol and phospholipid biosynthetic processes (Figure 3C). Interestingly, SREBF1 (also named SREBP1), FASN, and SCD1 expression was significantly downregulated after ZFAS1 knockdown compared with the control (Figure 3A). Consistently, qRT-PCR and western blot analysis confirmed that knockdown of ZFAS1 could downregulate SREBP1, FASN, and SCD1 expression in CRC cells (Figure 3D). Based on these findings, we hypothesized that ZFAS1 could facilitate the accumulation of lipids by regulating SREBP1 to promote the malignant phenotype of CRC cells. To validate this assumption, we first measured the content of three typical lipids (triglycerides and cholesterol) in peripheral blood of CRC patients and healthy controls. The results showed that the levels of triglycerides and cholesterol are lower in CRC patients’ peripheral blood compared with healthy controls (Table S3). Moreover, knockdown of ZFAS1 could decrease the content of lipids in SW480 and RKO cells (Figures 3E–3G). Taken together, ZFAS1 may regulate SREBP1 to facilitate the enrichment of lipids and thus promote the malignant phenotype of CRC cells.

### ZFAS1 cooperates with PABP2 to maintain SREBP1 mRNA stability

**Figure 4:**
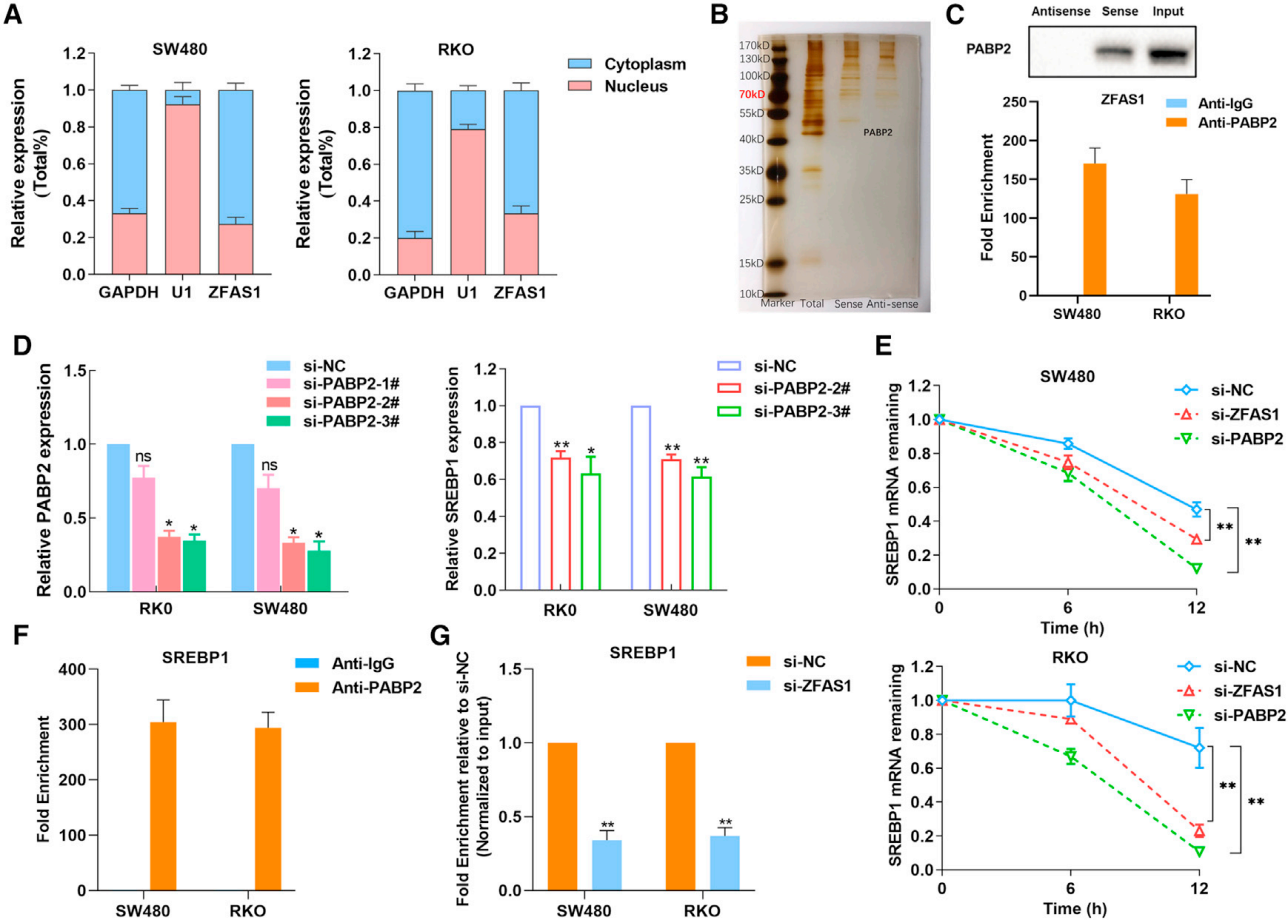
ZFAS1 cooperates with PABP2 to stabilize SREBP1 mRNA (A) Nuclear and cytoplasmic RNA fractions were isolated from SW480 and RKO cells. The distribution of ZFAS1 was examined by qRT-PCR. GAPDH was used as a cytoplasmic control, and U1 was used as a nuclear control. (B) RNA pulldown assays were performed with biotin-labeled sense or antisense (negative control) full-length ZFAS1 RNA in SW480 cells. Precipitated proteins were subjected to SDS-PAGE and stained with silver. (C) Protein levels in immunoprecipitates were determined by western blot assay. The expression level of PABP2 protein is presented. RIP experiments were performed in SW480 and RKO cells, and the precipitated RNA was subjected to qRT-PCR analysis of ZFAS1. The fold enrichment of ZFAS1 in PABP2 is relative to the IgG control. (D) The PABP2 and SREBP1 mRNA expression levels in SW480 and RKO cells transfected with PABP2 siRNAs or negative control siRNA were examined by qRT-PCR. (E) SW480 and RKO cells were transfected with ZFAS1, PABP2, or negative control siRNA. Forty-eight hours later, the cells were incubated with actinomycin D (2.5 μg/mL) for the indicated periods of time. The SREBP1 mRNA levels were then analyzed by real-time RT-PCR. (F) RIP experiments were performed in SW480 and RKO cells, and the precipitated RNA was subjected to qRT-PCR analysis of SREBP1. The fold enrichment of SREBP1 in PABP2 is relative to the IgG control. (G) RIP experiments were performed in SW480 and RKO cells transfected with ZFAS1 or negative control siRNA, and the precipitated RNA was subjected to qRT-PCR analysis of SREBP1. Three independent experiments were performed. Data are shown as the mean ± SD. ∗p < 0.05, ∗∗p < 0.01.

We next explored how ZFAS1 increased SREBP1 expression. First, we evaluated the distribution of ZFAS1 in SW480 and RKO cells and found that ZFAS1 was mainly enriched in the cytoplasm (Figure 4A). In the cytoplasm, lncRNAs can interact with proteins or RNAs to influence protein activity or the localization of protein complexes. Moreover, they can also act as “miRNA sponges” that affect the ceRNA network. Because many studies have confirmed that ZFAS1 could act as an miRNA sponge to regulate the expression of target genes, we next explored whether ZFAS1 can directly interact with proteins to exert regulatory functions. RNA pulldown assays were performed, and proteins were subjected to SDS-PAGE, stained with silver, and determined by mass spectrometry (Figure 4B; Table S4). We found that ZFAS1 could bind with polyadenylate-binding protein 2 (PABP2) (with 16 peptides) in CRC cells, which has not been reported. Furthermore, we confirmed their interaction in CRC cells by western blot following RNA pulldown and RIP assays (Figure 4C). In addition, overexpression of PABP2 could partially reverse downregulation of ZFAS1-induced inhibition of cell proliferation and target gene expression (Figures S1A–S1D).

PABP2 is able to stabilize mRNA by binding to poly(A) and to poly(G) with high affinity in 3′-untranslated regions (UTRs).^29^ Then, we performed a gene microarray to explore the PABP2-regulated genes in CRC cells, and we found that downregulation of PABP2 also decreased SREBP1 expression (Table S2). To further evaluate whether PABP2 could regulate SREBP1 expression, we first downregulated PABP2 expression with siRNAs. Then, the qRT-PCR results showed that knockdown of PABP2 decreased SREBP1 expression in CRC cells (Figure 4D). To verify that ZFAS1 and PABP2 could affect the stability of SREBP1 mRNA, SW480 and RKO cells with knockdown of ZFAS1 or PABP2 were treated with actinomycin D for different periods of time to measure the decay of SREBP1 mRNA. The results showed that knockdown of ZFAS1 and PABP2 resulted in a decrease in the half-life of SREBP1 mRNA (Figure 4E). Moreover, the RIP assay revealed that PABP2 can bind with SREBP1 mRNA in CRC cells, indicating that ZFAS1 and PABP2 could stabilize SREBP1 mRNA (Figure 4F). To further determine whether PABP2 promotes SREBP1 mRNA stability by interacting with ZFAS1, we performed an RIP assay in ZFAS1-downregulated and control cells. The results showed that downregulation of ZFAS1 reduced the interaction between PABP2 and SREBP1 mRNA in CRC cells (Figure 4G). These data suggest that ZFAS1 cooperates with PABP2 to stabilize SREBP1 mRNA.

### ZFAS1 promotes the malignant phenotype of CRC partially by regulating SREBP1

**Figure 5:**
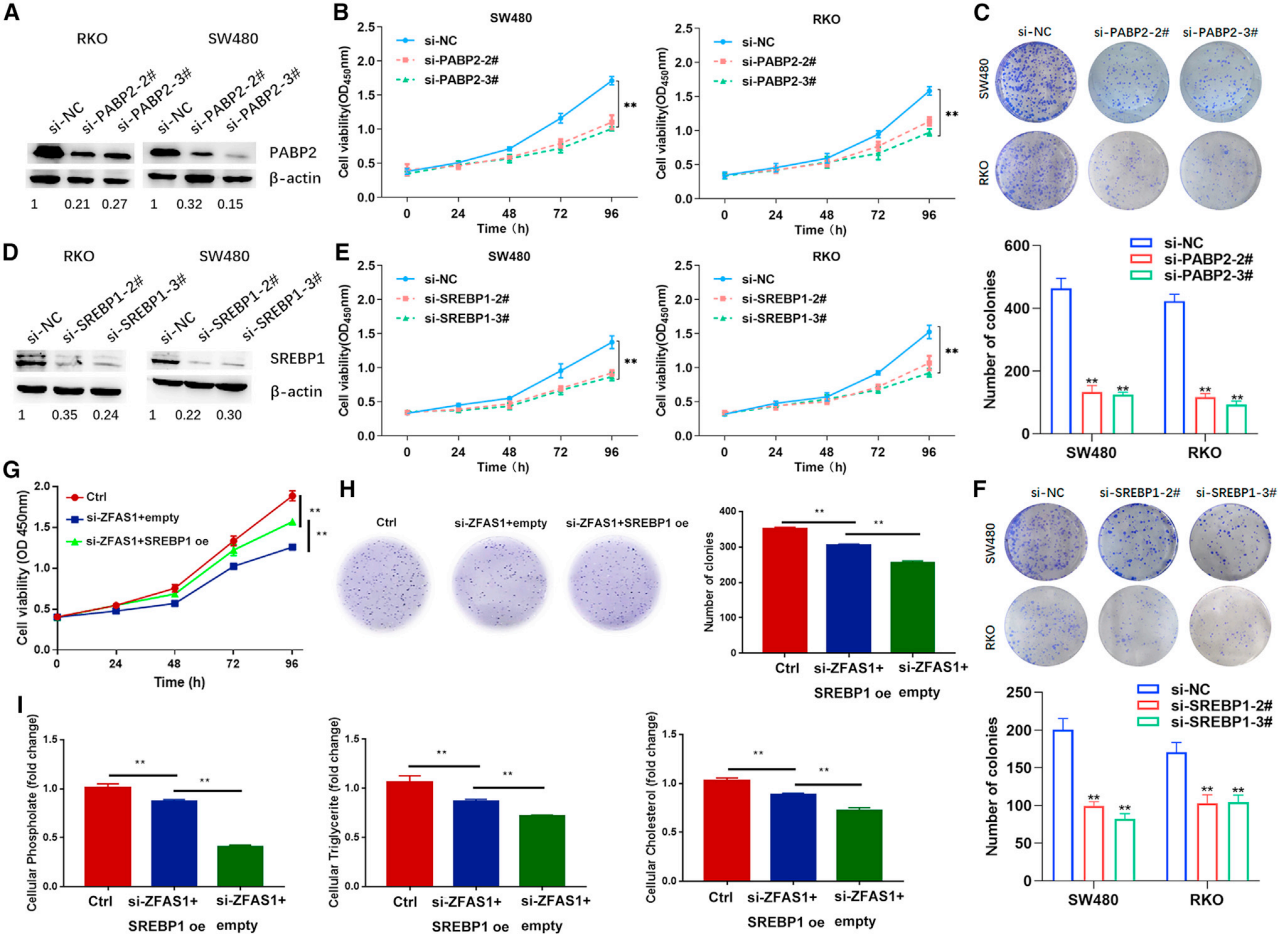
ZFAS1 contributes to CRC cell proliferation and lipogenesis by regulating SREBP1 expression (A) The PABP2 protein levels in SW480 and RKO cells transfected with PABP2 siRNAs or negative control siRNA were examined by western blot. (B) The CCK-8 assay showed that knockdown of PABP2 inhibited the growth of SW480 and RKO cell lines. (C) The colony formation assay showed that the colony numbers of the si-PABP2 groups were less than those of the control group. (D) The SREBP1 protein levels in SW480 and RKO cells transfected with SREBP1 siRNAs or negative control siRNA were examined by western blot. (E) The CCK-8 assay showed that knockdown of SREBP1 inhibited the growth of SW480 and RKO cell lines. (F) The colony formation assay showed that the colony numbers of the si-SREBP1 groups were less than those of the control group. (G) The CCK-8 assay showed that rescue of SREBP1 partially reversed the effect of ZFAS1 downregulation on the proliferation of CRC cells. (H) The colony formation assay showed that rescue of SREBP1 partially reversed the effect of ZFAS1 downregulation on colony formation of CRC cells. (I) Cellular lipids were detected by relative kits, and the results showed that rescue of SREBP1 partially reversed the effect of ZFAS1 downregulation on lipogenesis in SW480 cells. Three independent experiments were performed. Data are shown as the mean ± SD. ∗∗p < 0.01.

To further investigate the role of PABP2 and SREBP1 in CRC cells, we performed loss-of-function assays. As shown in Figures 5A and 5B, two siRNAs significantly knocked down PABP2 protein expression, and downregulation of PABP2 inhibited CRC cell proliferation. Consistently, knockdown of PABP2 also impaired CRC cell colony formation ability (Figure 5C). Moreover, downregulation of SREBP1 with two different siRNAs also repressed CRC cell proliferation and colony formation (Figures 5D–5F). To determine whether ZFAS1 exerts an oncogenic function dependent on maintaining SREBP1 expression in CRC cells, we performed rescue experiments. As shown in Figures 5G, 5H, and S1E, overexpression of SREBP1 partially reversed the ZFAS1 downregulation-mediated inhibition of CRC cell proliferation and colony formation. In addition, overexpression of SREBP1 partially rescued the effect of si-ZFAS1 on lipogenesis in CRC (Figure 5I). These findings indicate that ZFAS1 exerts its oncogenic function in CRC cells and might be partly dependent on the regulation of SREBP1 expression.

### Downregulation of ZFAS1 impairs CRC cell tumor growth and invasion *in vivo*

**Figure 6:**
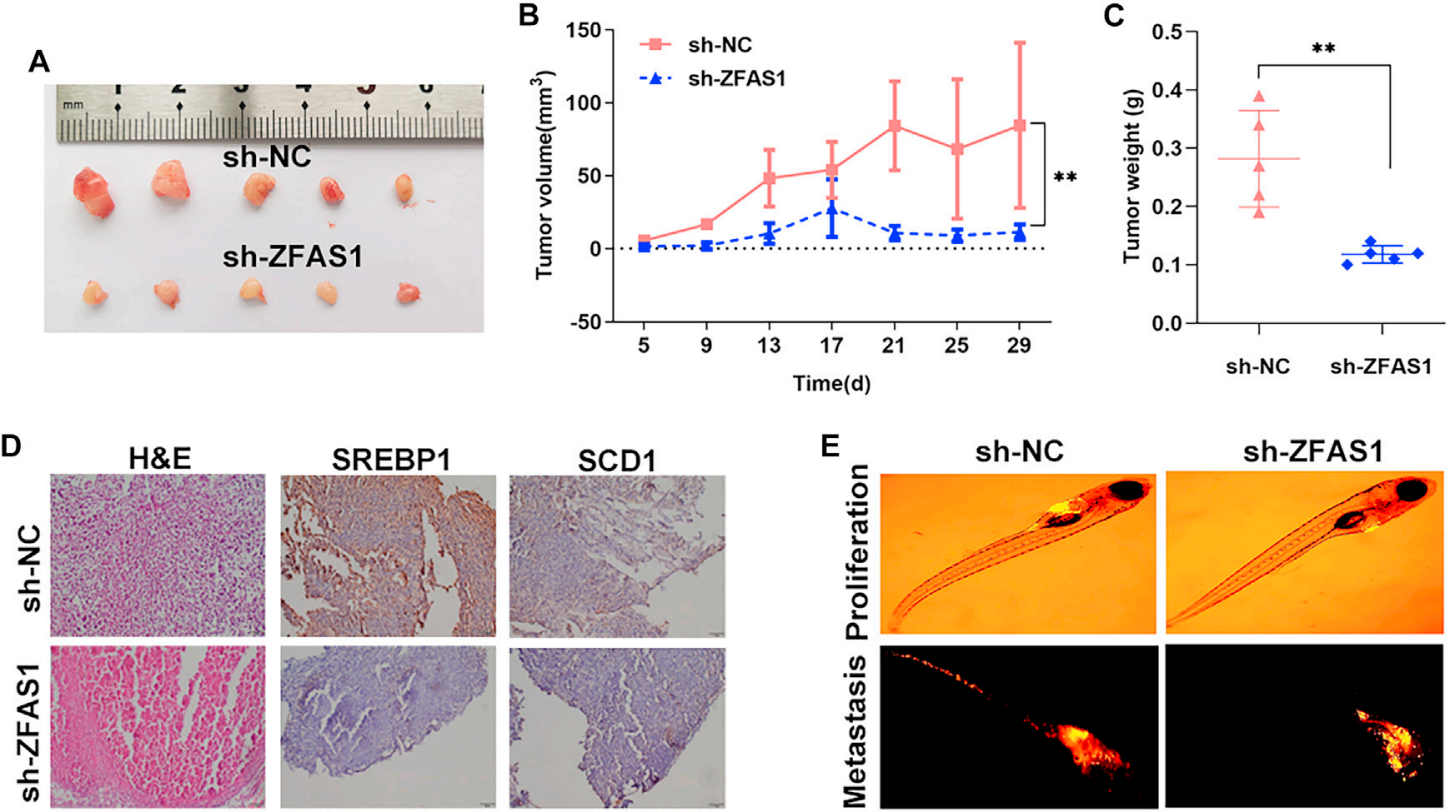
ZFAS1 promotes the malignant phenotype of CRC *in vivo* (A) Whole tumor tissues collected from two groups of mice treated with ZFAS1 or negative control shRNA-transfected SW480 cells are shown. (B) The volumes of tumors from two groups of mice treated with ZFAS1 or negative control shRNA-transfected SW480 cells are shown. (C) The tumor weights from two groups of mice treated with ZFAS1 or negative control shRNA-transfected SW480 cells are shown. (D) Immunohistochemistry staining of human colorectal cancer tissues with SREBP1 and SCD1 antibodies is shown. (E) CRC cells transfected with ZFAS1 or negative control shRNA were injected into the PVS of 2-dpf wild-type zebrafish larvae. Images were taken using a stereomicroscope at 4 dpi. CM-DiI-positive areas in the yolk were quantified for proliferation, and CM-DiI-positive areas in the trunk were quantified for metastasis. The regions enclosed by the red dashed curve in the segmented images were selected for calculating tumor areas in the yolk or trunk. ∗∗p < 0.01.

A xenograft tumor model was used to explore the oncogenic role of ZFAS1 *in vivo*. SW480 cells transfected with ZFAS1 or negative control small hairpin RNA (shRNA) were injected intramuscularly into the left hindlimb muscle of mice, and tumor nodules were harvested at 4 weeks after injection (Figure 6A). Silencing ZFAS1 significantly inhibited tumor growth (weight and volume) *in vivo* (Figures 6B and 6C). Meanwhile, the results of immunohistochemistry staining in tumor tissues collected from the mice showed that downregulation of ZFAS1 led to decreased protein levels of SREBP1 and SCD1, which is consistent with the *in vitro* findings. Next, a zebrafish xenograft model based on stereomicroscopy was used to verify the effect of ZFAS on CRC cell proliferation and metastasis *in vivo*. We quantified the area with CM-DiI-positive signals, which represented the tumor area in the yolk and trunk, compared with the control group. We found that the CM-DiI-positive area was significantly smaller in the yolk and trunk when ZFAS1 was knocked down in SW480 cells, indicating that downregulation of ZFAS1 decreased the proliferation (in yolk) and metastasis (in trunk) of SW480 cells (Figure 6D).

## Discussion

Abnormal lipid metabolism is one of the characteristics of early abnormalities in tumor cells, and lipid synthesis can not only provide a large number of membrane phospholipids for the continuous division and proliferation of tumor cells but also yield a series of cancer-promoting lipid signaling molecules, such as phosphatidylinositol and sphingomyelin.^7^ Recent advances have revealed that lncRNAs play a vital role in cell metabolism by regulating the reprogramming of metabolic pathways in cancer cells. lncRNAs can regulate various metabolic enzymes that integrate cell malignant transformation and metabolic reprogramming.^30^ For example, Cui et al. revealed that lncRNA-HULC could modulate lipogenesis in hepatocellular carcinoma by regulating the miR-9/PPARA/ACSL1 pathway.^24^ In human nasopharyngeal carcinoma cells, knockdown of HOTAIR causes a decrease in free fatty acids and FASN at the transcriptional and posttranscriptional levels.^31^

ZFAS1 has been demonstrated to be an oncogenic lncRNA in the occurrence and development of multiple cancers, including CRC.^32^ For instance, Wei etc. reported that ZFAS1 promoted CRC cells proliferation and migration through accelerating SNORD12C and SNORD78-mediated rRNA-2′-O-methylation via recruiting NOP58 (PMID: 32443980), or by interacting with DDX21 to regulate POLR1B expression (PMID: 33202381). However, most recent studies have mainly revealed that ZFAS1 participates in the multilayered biological functions of cancer cells, such as proliferation, metastasis, and chemoresistance, by regulating miRNAs.^33^^,^^34^ Here, we revealed a novel role and underlying molecular mechanism of ZFAS1 in CRC cells. In this study, we found that the expression of ZFAS1 was higher in both CRC tissues and cell lines, and ZFAS1 overexpression was positively correlated with advanced pathological stages and larger tumor sizes. Furthermore, knockdown of ZFAS1 significantly suppressed the proliferation of CRC cells *in vitro* and *in vivo*, which was consistent with previous studies. Then, we performed RNA sequencing analysis to identify the downstream target genes and surprisingly found that ZFAS1 was able to affect steroid biosynthesis and fatty acid metabolism. Importantly, how ZFAS1 affects lipid metabolism and the underlying mechanism have not been demonstrated.

Among these differentially expressed genes after ZFAS1 knockdown, we found that SREBP1 was the most downregulated gene. SREBP1 is a master regulator of lipid homeostasis, which preferentially activates genes for fatty acid synthesis, such as SCD1 and FASN.^35^ Consistently, our data also showed that SCD1 and FASN expression was decreased following ZFAS1 downregulation in CRC cells. Because many lncRNAs exert their functions through interaction with their target proteins, we conducted an RNA pulldown assay combining mass spectrometry analysis. However, we did not identify the SREBP1 protein, which indicates that ZFAS1 may indirectly regulate SREBP1 expression. A recent study reported that H19 facilitates polypyrimidine tract-binding protein 1 (PTBP1) binding to SREBP1 mRNA in the cytoplasm, resulting in increased stability, which in turn augments SREBP1 transcriptional activity.^36^ Interestingly, we found that ZFAS1 could bind with polyadenylate-binding protein 2 (PABP2), which can also maintain RNA stability by binding with high affinity to poly(A) tails. Furthermore, we found that downregulation of ZFAS1 and PABP2 both decreased the mRNA stability of SREBP1 in CRC cells, and silencing of ZFAS1 could reduce the interaction between PABP2 and SREBP1 in CRC cells. Finally, we confirmed that knockdown of PABP2 and SREBP1 also impaired CRC cell proliferation as well as downregulation of ZFAS1, and rescue experiments confirmed that overexpression of SREBP1 could partially reverse the effect of ZFAS1 silencing on CRC cell lipogenesis and proliferation. These findings revealed that ZFAS1 promotes CRC cell lipogenesis in a manner partially dependent on the regulation of SREBP1 expression by binding to PABP2.

In conclusion, our study provides evidence that disorders of fat metabolism are important in the development and progression of CRC, and overexpression of ZFAS1 is associated with disease progression in CRC patients. ZFAS1 reprograms fat metabolism to promote CRC progression by binding with PABP2 to facilitate the interaction between PABP2 and SREBP1, thereby stabilizing SREBP1 mRNA and activating its downstream genes SCD1 and FASN. Consequently, ZFAS1 is considered a promising biomarker for CRC diagnosis and prognosis and could be a potential therapeutic target for CRC treatment.

## Materials and methods

### Bioinformatics analysis

The GSE5206,^37^ GSE21510,^38^ and GSE9348^39^ datasets were downloaded from the GEO database (https://www.ncbi.nlm.nih.gov/). The expression of ZFAS1 in cancer tissues compared with normal tissues from TCGA dataset was analyzed by GEPIA (http://firebrowse.org). The relationship between the expression of ZFAS1 and invasive status and microsatellite stability in CRC were analyzed by Tanric (https://ibl.mdanderson.org/tanric/_design/basic/main.html). For Kyoto Encyclopedia of Genes and Genomes (KEGG) pathway and Gene Ontology analysis, a total of 2,165 differentially expressed genes were analyzed with DAVID software (https://david.ncifcrf.gov/home.jsp). GSEA was performed using the website https://www.gsea-msigdb.org/gsea/index.jsp.

### Clinical samples and cell lines

CRC and adjacent tissues (n = 30) were obtained by surgical operations from the Second Affiliated Hospital of Nanjing Medical University from June 2017 to July 2019. Tumor staging was confirmed on the basis of the eighth edition of the American Joint Committee on Cancer (AJCC) TNM Staging Manual (eighth edition, 2017). Peripheral blood samples were obtained from 218 patients with CRC and 238 healthy people at Zhongda Hospital, Medical School, Southeast University (Nanjing, China). This study was approved by the Review Board of Zhongda Hospital, Medical School, Southeast University, and Second Affiliated Hospital of Nanjing Medical University. All patients provided signed informed consent. CRC cell lines (SW480, RKO, HT-29, HCT116) and human normal colonic epithelial cells (FHCs) were obtained from the Institute of Biochemistry and Cell Biology of the Chinese Academy of Sciences (ICBC, Shanghai, China). SW480, RKO, HT-29, and HCT116 cell lines were cultured in DMEM (Gibco, Thermo Fisher, United States), while FHC was performed in RPMI-1640 (Gibco, Thermo Fisher, United States) containing 10% fetal bovine serum (FBS, ScienCell, Carlsbad, CA), 1% streptomycin (HyClone), and 1% penicillin (HyClone) in a cell incubator (37°C, 5% CO_2_).

### RNA extraction and qRT-PCR

TRIzol reagent (Invitrogen, Carlsbad, United States) was used to isolate the total RNA from the cultured cells and tissue samples. The reverse transcription reaction of total RNA was conducted using the PrimeScript RT reagent kit with gDNA Eraser (Takara, RR047A, Japan). The obtained cDNA was subjected to PCR amplification using SYBR green (Takara, RR420A, Japan) with an ABI 7500 Real-Time PCR System (Thermo Fisher). β-Actin was used as an endogenous control, and the 2^−ΔΔCt^ method was used to calculate the relative expression. Primers sequences are listed in Table S1.

### Isolation of cytoplasmic and nuclear RNA

The distribution of ZFAS1 in the cytoplasmic and nuclear fractions of SW480 and RKO cells was evaluated with the PARIS Kit (Life Technologies, United States) according to the manufacturers' instructions. Briefly, cells were washed twice using prechilled PBS and then lysed. After centrifugation at 300 × *g* for 5 min, the supernatant (cytoplasmic fraction) was collected, and the remaining pellet, which was considered to be the nuclear fraction, was collected after an additional five washes using PBS. The experiment was repeated at least three times.

### Transfection

The siRNAs targeting ZFAS1 were purchased from RiboBio (Guangzhou, China). The full-length cDNA of human SREBP1 was PCR amplified and cloned into the expression vector pcDNA3.1 (Xinbeke Biosciences, Shandong, China). When the cells in six-well plates were approximately 40% confluent, siRNAs (or plasmids) were transfected into cells using Lipofectamine 3000 (Thermo Fisher, United States) according to the product instructions. All the mentioned sequences are listed in Table S1.

### CCK-8 and colony formation assays

All cell proliferation assays were performed 24 h after transfection. For the Cell Counting Kit-8 (CCK-8, Dojindo, Japan) assay, cells were plated in 96-well plates at a density of 1,000 cells/100 μL, and the absorbance at 450 nm was measured every 24 h. For the colony formation assay, cells were seeded into six-well plates at 1,000 cells/well and cultured with complete medium for 10 days. Then, the cells were stained with a 10% crystal violet solution (Sigma, NY). Visible colonies were counted. All the assays were repeated three times.

### Transwell assays

Transwell invasion assays were performed in 24-well plates (Corning, MA) using a 6.5-mm diameter Transwell chamber with an 8-μm pore polycarbonate membrane insert (Corning). After 48 h of transfection, SW480 or RKO cells were plated on the upper chambers coated with 50 μL of Matrigel (Corning) in serum-free medium. After incubation for 24 h at 37°C, cells were fixed with 4% paraformaldehyde, stained with crystal violet solution, and counted at 200× magnification under a microscope. The assay was repeated three times. The numbers of cells counted in five random fields were averaged.

### Flow cytometric analysis of cell-cycle distribution

For cell-cycle analysis, the cells were harvested and fixed with 70% ethanol for 12 h at −20°C. Then, 0.5 mL of FxCycle PI/RNase Staining Solution was added to each sample. After incubation for 15–30 min at room temperature in the dark, the samples were analyzed by a FACSCalibur flow cytometer. All samples were assayed in triplicate.

### Quantification of cholesterol, triglyceride, and phospholipid

For quantitative estimation of triglycerides or phospholipids in CRC cells, an enzymatic assay was performed using EnzyChrom Triglyceride Assay Kits (BioAssay Systems, Hayward, CA) and EnzyChrom Phospholipid Assay Kits (BioAssay Systems, Hayward, CA) according to the manufacturer's protocols. Cellular cholesterols were assayed using a total tissue cholesterol kit (E1015-105, Applygen Technologies, Beijing, China).

### Western blot analysis

Total cellular protein was extracted using RIPA lysis buffer containing protease inhibitor cocktail (Beyotime, China). The extracted proteins were separated by SDS-PAGE and transferred onto polyvinylidene fluoride (PVDF) membranes (Millipore, NY), which were subsequently blocked with a 5% solution of nonfat milk for 1 h. The membranes were then incubated with primary antibodies at 4°C overnight, followed by secondary antibody incubation for 1 h at room temperature. The proteins were visualized with a SuperLumia ECL HRP Substrate Kit (Millipore, NY) and detected using an LAS4000 chemiluminescence detection system (Fuji, Tokyo, Japan). The primary antibodies used were as follows: anti-β-actin (1:1,000, AF5001, Beyotime), SREBP1 (1:1,000, 14088-1-AP, Proteintech), and SCD1 (1:500, sc-515844, Santa Cruz Biotechnology). The anti-β-actin antibody was used as an internal control.

### RNA pulldown assay

The pulldown assay with biotinylated RNA was performed using the Pierce Magnetic RNA-Protein Pull-Down Kit (Thermo Fisher). Briefly, ZFAS1 RNAs were transcribed *in vitro* using T7 RNA polymerase (Ambio Life, Austin, TX), purified using the RNeasy Plus Mini Kit (Qiagen), and treated with RNase-free DNase I (Qiagen). Transcribed RNAs were biotin labeled with Biotin RNA Labeling Mix (Ambio Life). Positive, negative, and biotinylated RNAs were mixed and incubated with SW480 cell lysates. Magnetic beads were added to each binding reaction, followed by incubation at room temperature. Then, the beads were washed with washing buffer. The eluted proteins were detected by mass spectrometry and western blotting.

### RIP assay

RIP assays were performed with a Magna RIP RNA-Binding Protein Immunoprecipitation Kit (Millipore, Billerica, MA) in accordance with the manufacturer's protocol. The relative expression of ZFAS1 and SREBP1 mRNA was analyzed by qRT-PCR. The RIP fraction Ct value was normalized to the immunoglobulin (Ig) G RNA fraction Ct value.

### RNA stability assay

Forty-eight hours after transfection, SW480 and RKO cells were incubated with actinomycin D (2.5 μg/mL) for 6 h and 12 h, and groups without actinomycin D treatment were used as negative controls. Total RNA was then extracted and analyzed by qRT-PCR to examine SREBP1 mRNA stability.

### Tumor formation assay in a nude mouse model

All animal studies were conducted in accordance with NIH animal use guidelines, and protocols were approved by the Nanjing Medical University Animal Care Committee. Before tumor transplantation, SW480 cells were transfected with ZFAS1 shRNAs. Transfection was performed by transient transfection according to the specifications of Lipofectamine 3000 (Invitrogen, Carlsbad, CA). sh-NC was used as a control, and a total of 10 μg plasmid vectors were transfected into cells for each group. The shRNA sequences are shown in Table S4. We used seven mice per group, and 2 × 10^6^ CRC tumor cells in 100 μL of sterile PBS were injected subcutaneously into each mouse at the age of 6–8 weeks. Tumors were harvested at 6 weeks after injection. The weight of the tumor was measured on the scale, and the tumor volume was estimated using calipers ([length × width^2^]/2).

### Zebrafish xenograft model

Adult zebrafish and embryos were cultured according to the breeding conditions. The zebrafish AB wild type was used in our study. Zebrafish handling procedures were approved by Nanjing Medical University. Cultured cancer cells were labeled with CM-DiI (Invitrogen, United States) before injection. The cells were then examined via fluorescence microscopy. Subsequently, 2-day-postfertilization (dpf) zebrafish larvae were mounted using a 1.2% low-melting gel (Promega, United States), and then approximately 400 CM-DiI-labeled cells were injected into the perivitelline space (PVS) of each larva under a microinjector (Picosprizer III, United States). After injection, the xenografts were cultured at 34°C. At 24 h post injection (hpi), zebrafish larvae with similar sizes of transplanted cells were collected for further analysis and then cultured at 34°C until the end of experiments. At 4 days post injection (dpi), the zebrafish larvae were also mounted using a 1.2% low-melting gel for the imaging experiments. Imaging experiments were performed via a stereomicroscope (MVX10, Olympus, Japan). The spatial resolution of the images was 1,600 × 1,200 (MVX10).

### Immunohistochemistry

Human colorectal cancer tissue sections were deparaffinized and rehydrated through graded alcohol. Endogenous peroxidase activity was blocked by incubation in 3% H_2_O_2_, and antigen retrieval was carried out with 0.01 M citrate buffer (pH 6.0). Immunostaining was performed using the following antibodies: anti-SREBP1 (1:100, Proteintech), anti-SCD1 (1:50, Santa Cruz Biotechnology), and anti-Ki-67 (1:300, Nakasugi Bridge).

### Statistical analysis

Stata 13.0, GraphPad Prism 7.0, and ImageJ were used for image editing and data analysis, and t tests were performed to compare quantitative data. Classification data were compared using the chi-square test, and p < 0.05 was considered statistically significant (∗p < 0.05, ∗∗p < 0.01, ns, not significant).

## Availability of data and materials

The data supporting the conclusions of this article are presented within the article and its additional files.

## Ethics approval and consent to participate

All animal experiments were approved by the Institutional Animal Care and Use Committee of Nanjing Medical University. The human tissue study was approved by the Medical Ethics Committee of Second Affiliated Hospital of Nanjing Medical University and Zhongda Hospital, Medical School, Southeast University. Written informed consent was obtained from all participants.

## Supplemental information

Document S1. Figure S1 and Tables S1 and S3

Table S2. Gene profiling of CRC cells following downregulation of ZFAS1 or PABP2

Table S4. ZFAS1-interacting proteins in CRC cells that were determined by MS

Document S2. Article plus supplemental information
