# Supplementary material for: The lncRNA ZFAS1 regulates lipogenesis in colorectal cancer by binding polyadenylate-binding protein 2 to stabilize SREBP1 mRNA
Source: Mol Ther Nucleic Acids. 2021 Dec 11;27:363–74. doi: 10.1016/j.omtn.2021.12.010 (PMC8728310; doi:10.1016/j.omtn.2021.12.010)
Supplement: Document S1. Figure S1 and Tables S1 and S3 [file mmc1.pdf]

OMTN, Volume *27*

Supplemental information

The lncRNA ZFAS1 regulates lipogenesis in colorectal cancer by binding polyadenylate-binding protein 2 to stabilize SREBP1 mRNA

Huishan Wang, Yuli Chen, Yanwen Liu, Qiuhui Li, Jing Luo, Li Wang, Yuanyuan Chen, Chen Sang, Wen Zhang, Xianxiu Ge, Zhifeng Yao, Lin Miao, and Xianghua Liu

Supplementary Figure 1. ZFAS1 cooperates with PABP2 in CRC cell. (A, B), qPCR and western blot analysis of SREBP1 and SCD1 expression levels in SW480 cell transfected with negative control, ZFAS1 siRNA, or co-transfected with ZFAS1 siRNA and PABP2 over-expression plasmid. (C, D), The CCK-8 and colony formation assays showed cell viability and colony formation ability of SW480 cell transfected with negative control, ZFAS1 siRNA, or co-transfected with ZFAS1 siRNA and PABP2 over-expression plasmid. (E), Western blot analysis of SREBP1 expression level in SW480 cell transfected with negative control, ZFAS1 siRNA, or co-transfected with ZFAS1 siRNA and SREBP1 over-expression plasmid.

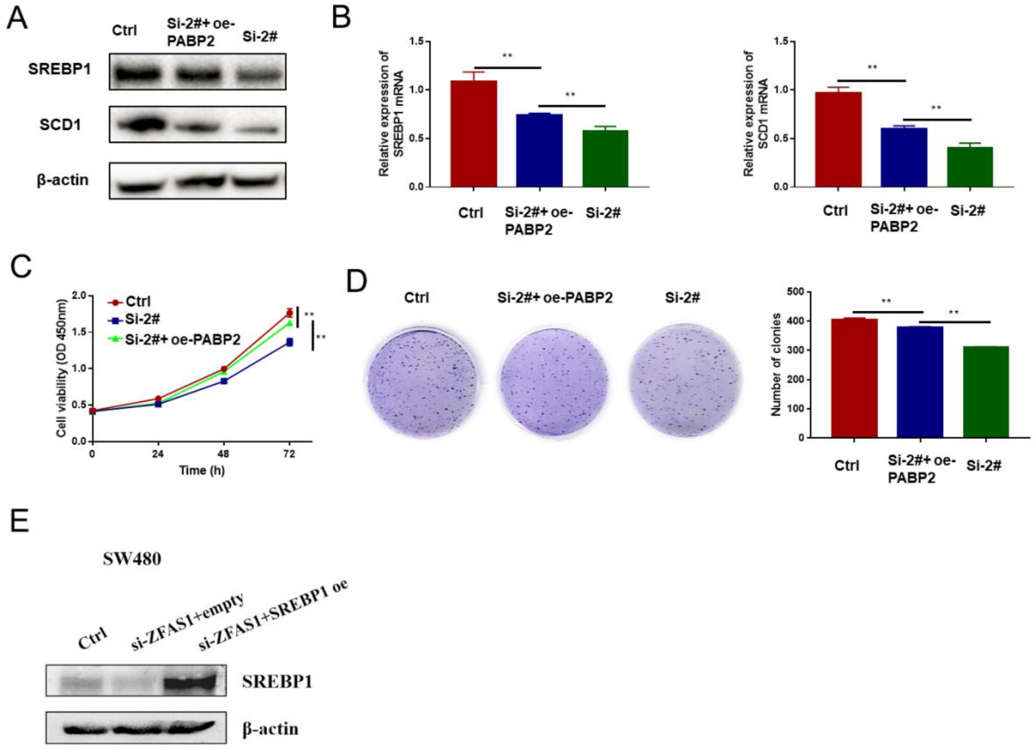

Supplementary Table.1 Sequence of primers, siRNAs and shRNAs that used in this study.

**Primers**	Forward primer
β-actin F	TGGCACCCAGCACAATGAA
β-actin R	CTAAGTCATAGTCCGCCTAGAAGCA
ZFAS1 F	ACGTGCAGACATCTACAACCT
ZFAS1 R	TACTTCCAACACCCGCAT
U1 F	GAGGCTTATCCATTGCACTCC
U1 R	CCCACTACCACAAATTATGCAG
SREBP1 F	GGAGCCATGGATTGCACATT
SREBP1 R	CAGGAAGGCTTCCAGAGAGG
FASN F	AAGGACCTGTCTAGGTTTGATGC
FASN R	TGGCTTCATAGGTGACTTCCA
SCD1 F	CCCCACCTACAAGGATAAGGA
SCD1 R	CACGAGCCCATTCATAGACAT
GAPDH F	GAAGGTGAAGGTCGGAGTC
GAPDH R	GAAGATGGTGATGGGATTTC
PABP2 F	GGAGCTGGAAGCTATCAAAGC
PABP2 R	CCTGGAGGTGGACTCATATTCA
**siRNA**	
PABP2（PABPN1)siRNA #1	CAGATGAATATGAGTCCACCT
PABP2（PABPN1)siRNA #2	CCTTAGATGAGTCCCTATTTA
PABP2（PABPN1)siRNA #3	TAGAGCGACATCATGGTATTC
SREBP1 siRNA #1	TATTCCGGGAACATCTCTTAG
SREBP1 siRNA #2	AGACATGCTTCAGCTTATCAA
SREBP1 siRNA #3	TGAGGCTCCTGTGCTACTTTG
ZFAS1-siRNA #1	AAGUGAAGAUCUGGCUGAACCAGUU
ZFAS1-siRNA #2	GCCCACUUCAAGAAUGUCAUUGUUA
Si-NC	UUCUCCGAACGUGUCACGUTT
**shRNA**	
sh-ZFAS1	GATTCAGTCTGCCTTGTAACA

Supplementary Table.2 Gene profiling of CRC cells following down-regulation of ZFAS1 or PABP2.

Supplementary Table 3. Analysis of Lipid Metabolism in Patients with Colorectal Cancer and Normal Physical Examination

Group	N	Triglycerides mmol/L	Cholesterol mmol/L	High density cholesterol mmol/L	Low density cholesterol mmol/L
Control	238	1.51(1)	4.94(1.21)	1.25(0.44)	3.295(1.22)
CRC patient	218	1.37(0.87)	4.54(1.45)	1.125(0.44)	2.74(1.3)
Z		-1.222	-4.619	-4.986	-4.938
p		＞0.05	＜0.05	＜0.05	＜0.05

Supplementary Table 4. ZFAS1 interacted proteins in CRC cells that were determined by MS
